# Genomic epidemiology and emergence of SARS-CoV-2 variants of concern in the United Arab Emirates

**DOI:** 10.1038/s41598-022-16967-w

**Published:** 2022-08-29

**Authors:** Habiba Alsafar, Mohammed Albreiki, Mira Mousa, Syafiq Kamarul Azman, Hema Vurivi, Fathimathuz Waasia, Dymitr Ruta, Farida Alhosani, Shereena Almazrouei, Rowan Abuyadek, Francis Selvaraj, Irene Chaves-Coira, Val Zvereff, Mohamed A. Y. Abdel-Malek, Nawal Alkaabi, Maimunah Uddin, Tayba Al Awadhi, Nada Al Marzouqi, Fatma Al Attar, Safeiya Al Shamsi, Fatima Al Shehhi, Hala Alteneiji, Kalthoom Mohamed, Noor Al Muhairi, Hussain AlRand, Asma Fikri, Andreas Henschel

**Affiliations:** 1 Center for Biotechnology, Khalifa University of Science and Technology, PO BOX, 127788 Abu Dhabi, United Arab Emirates; 2 Department of Biomedical Engineering, College of Engineering, Khalifa University of Science and Technology, Abu Dhabi, United Arab Emirates; 3 Emirates Bio-Research Center, Ministry of Interior, Abu Dhabi, United Arab Emirates; 4 Nuffield Department of Women’s and Reproduction Health, Oxford University, Oxford, UK; 5 Department of Electrical Engineering and Computer Science, Khalifa University of Science and Technology, Abu Dhabi, United Arab Emirates; 6 Emirates ICT Innovation Center (EBTIC), Khalifa University of Science and Technology, Abu Dhabi, United Arab Emirates; 7 Abu Dhabi Public Health Center, Abu Dhabi Department of Health, Abu Dhabi, United Arab Emirates; 8 High Institute of Public Health, Alexandria University, Alexandria, Egypt; 9 Department Laboratory Medicine Services, Sheikh Khalifa Medical City, Abu Dhabi, United Arab Emirates; 10 Molecular and Genetics Department, UniLabs, Abu Dhabi, United Arab Emirates; 11 Department of Molecular Diagnostics, National Reference Laboratory, Abu Dhabi, United Arab Emirates; 12 Department of Pathology, College of Medicine and Health Sciences, Khalifa University of Science and Technology, Abu Dhabi, United Arab Emirates; 13 Molecular Biology Laboratory, Mediclinic Alnoor Hospital, Abu Dhabi, United Arab Emirates; 14 Clinical Pathology Department, Faculty of Medicine, Assiut University, Assiut, Egypt; 15 Department of Pediatric Infectious Disease, Sheikh Khalifa Medical City, Abu Dhabi, United Arab Emirates; 16 Ministry of Health and Prevention, Dubai, United Arab Emirates

**Keywords:** Genetic association study, Genome, Immunogenetics, Sequencing, Infectious diseases

## Abstract

Since the declaration of SARS-CoV-2 outbreak as a pandemic, the United Arab Emirates (UAE) public health authorities have adopted strict measures to reduce transmission as early as March 2020. As a result of these measures, flight suspension, nationwide RT-PCR and surveillance of viral sequences were extensively implemented. This study aims to characterize the epidemiology, transmission pattern, and emergence of variants of concerns (VOCs) and variants of interests (VOIs) of SARS-CoV-2 in the UAE, followed by the investigation of mutations associated with hospitalized cases. A total of 1274 samples were collected and sequenced from all seven emirates between the period of 25 April 2020 to 15 February 2021. Phylogenetic analysis demonstrated multiple introductions of SARS-CoV-2 into the UAE in the early pandemic, followed by a local spread of root clades (A, B, B.1 and B.1.1). As the international flight resumed, the frequencies of VOCs surged indicating the January peak of positive cases. We observed that the hospitalized cases were significantly associated with the presence of B.1.1.7 (p < 0.001), B.1.351 (p < 0.001) and A.23.1 (p = 0.009). Deceased cases are more likely to occur in the presence of B.1.351 (p < 0.001) and A.23.1 (p = 0.022). Logistic and ridge regression showed that 51 mutations are significantly associated with hospitalized cases with the highest proportion originated from S and ORF1a genes (31% and 29% respectively). Our study provides an epidemiological insight of the emergence of VOCs and VOIs following the borders reopening and worldwide travels. It provides reassurance that hospitalization is markedly more associated with the presence of VOCs. This study can contribute to understand the global transmission of SARS-CoV-2 variants.

## Introduction

Coronavirus disease 2019 (COVID-19) caused by severe acute respiratory syndrome coronavirus 2 (SARS-CoV-2) was identified in Wuhan, China, in late December 2019^1^. On 12 March 2020, WHO declared the ongoing SARS-CoV-2 outbreak as a pandemic, indicating a significant public health challenge^2^. To date (14 June 2022), over 535 million confirmed cases of COVID-19 with more than ~ 6 million deaths have been reported in 192 countries^3^. Due to its high fatality and transmission rate^4–6^, COVID-19 resulted in worldwide lockdown, closure of schools and businesses, and a huge burden on the healthcare system.

The United Arab Emirates (UAE) is an international tourist destination with Dubai as the major metropolitan travel hub in the Middle East. The first case of SARS-CoV-2 was reported on January 29th, 2020^3^ which had subsequently forced UAE public health authorities to adopt strict measures to contain the transmission as early as March 2020^7,8^. As a result of the second and third epidemic peaks, several high-throughput molecular projects such as the nationwide reverse transcription polymerase chain reaction (RT-PCR) screening project and surveillance of viral sequences were announced to extensively monitor the viral spread and early detection of infected patients. Tracking viral spread is being used to monitor mutations that might change the transmission, pathogenesis, or antigenic properties of the virus. Since the first SARS-CoV-2 genome sequencing on January 10th, 2020^9^, there have now been a substantial number of sequences of SARS-CoV-2 uploaded into a public database includes 2627 sequences from individuals in the UAE^10^.

Analysis of genomic sequences plays a major role in detecting the presence of SARS-CoV-2 variants of concern (VOCs), such as B.1.1.7 (alpha variant), B.1.351 (beta variant), and P.1 (gamma variant), which is associated with an increased viral transmission, pathogenicity, immune escape, and hospitalization in the latter part of 2020^11–13^. Similarly, variants of interest (VOIs), such as A.23.1, B.1.429 and B.1.525, appeared due to amino acid alterations associated with increased community transmission, and these variants have been detected in various countries. The earliest sequences of VOCs, published in the GISAID (Global Initiative on Sharing Influenza Data) database, in UAE was reported by Al Safar et al. (2021) with 19 cases of B.1.1.7 (EPI_ISL_859852) and 6 cases of B.1.351 (EPI_ISL_860088) indicating the emergence of VOCs in November 2020^10^. The common mutations in the previously reported VOCs were N501Y, E484K, E484Q, K417N, K417T, L452R and ∆69–70, which were associated with increased transmissibility, immune escape, and decreased neutralization^14–22^. The SARS-CoV-2 VOC and VOIs appear as a global threat throughout the world that hinders efforts to contain this pandemic.

Global massive ongoing transmission and the continuous development of new strains demonstrates that better mitigation measures are important to effectively control the spread of the virus. During the holiday season of late 2020, super-spreading events in the UAE, such as public gatherings, restaurants, weddings, and close living environments in hotels, contributed to the regional and national transmission of the virus. New variants of concerns have been introduced to the UAE in the last few months, instigating the spread of SARS-CoV-2, not only locally, but also globally via flight routes, ports, and trading movement.

Viral genomic sequencing is a fundamental technique to understand the dynamics of viral epidemic, epidemiological spread, transmission pattern, mutational spectrum, and evaluating countermeasures. The vaccination strategy of the UAE is leading globally, however the advent of new variants raised global public health concerns on the possible role of disease severity, immune escape, and antibody response. Therefore, this study aims to characterize the full genome sequence of SARS-CoV-2 between the period of 25 April 2020 to 15 February 2021 in the UAE to gain a deeper understanding of the molecular epidemiology and transmission pattern in the UAE. In addition, the present study aims to evaluate the association of VOCs and VOIs on patient clinical outcome and disease severity, as well as analyze the structural modifications of mutations in SARS-CoV-2.

## Methodology

### Ethics statement

This study has been approved by the local ethics committee at Abu Dhabi Health COVID-19 Research Ethics Committee (DOH/DQD/2020/538), SEHA Research Ethics Committee (SEHA-IRB-005) and Ministry of Health and Prevention (MOHAP/DXB-REC/ AAA/No. 80/2021). This study was conducted in accordance with international ethical standards (Declaration of Helsinki 1964) and UAE federal law No. (4) of 2016. Participant information was coded and held securely in compliance with the Data Protection Regulation of Khalifa University. Informed Consent was obtained from a family member of patients who were on ventilators with a signed agreement by a supervising physician. All data were de-identified prior to use.

### Study population and data collection period

This cross-sectional study recruited a total of 1,538 participants that have been tested positive for SARS-CoV-2 by quantitative real-time Polymerase Chain Reaction (qPCR) if the cycle threshold (Ct) value was 36 or less via nasopharyngeal swabs. Samples were collected between 25 April 2020 to 15 February 2021 from multiple sites across the seven emirates in the UAE (Abu Dhabi, Dubai, Sharjah, Ajman, Umm Al Quwain, Ras Al Khaimah, and Fujairah) from multiple medical centers, hospitals, quarantine camps and non-quarantine facilities (Supplementary Fig. 1). Extracted RNA from SARS-CoV-2 samples was amplified by WHO-recommended primers and probes targeting the ORF, N and S genes. Demographic and clinical data for SARS-CoV-2 sequenced samples in UAE are shown in Table 1. Due to the heterogeneous nature of COVID-19’s phenotype spectrum, a broad definition was utilized to categorize the severity status of the affected cases into home quarantine, hospitalized and deceased.

**Table 1:** Demographic of COVID-19 cases (n = 1274), stratified by VOC /VOI identification.

Variables	Total population % (n = 1274)	Non-VOC/VOI (n = 814) % (n)	VOC/VOI (n = 460) % (n)	P-value
**Gender**				
Male	59.0% (729)	62.1% (483)	53.8% (246)	0.004
Female	41.0% (506)	37.9% (295)	46.2% (211)	
**Age**				
< 15	10.5% (130)	27.2% (226)	22.8% (104)	0.006
16–28	15.5% (191)	26.7% (222)	23.6% (108)	
29–36	25.2% (311)	23.9% (198)	22.8% (104)	
37–47	23.4% (288)	22.2% (184)	30.9% (141)	
> 48	25.4% (313)			
**Nationality**				
Middle East	49.4% (488)	48.3% (299)	51.4% (189)	0.053
Asia	41.3% (408)	43.3% (268)	38.0% (140)	
Africa	4.2% (41)	4.5% (28)	3.5% (13)	
Europe	3.6% (36)	2.4% (15)	5.7% (21)	
America	1.4% (14)	1.5% (9)	1.4% (5)	
**Patient status**				
Home quarantine	81.0% (897)	86.7% (614)	70.9% (283)	< 0.001
Hospitalized	17.0% (188)	11.2% (79)	27.3% (109)	
Deceased	2.0% (22)	2.1% (15)	1.8% (7)	

### Library preparation and sequencing

Viral RNAs from COVID-19 patients were extracted using QIAamp Viral RNA Mini Kits (Qiagen, Hilden, Germany). RNA libraries from all samples were prepared for COVID sequencing using Illumina CovSeq Test and 8 IDT for Illumina-PCR indexes (San Diego, CA, USA), following the manufacturer’s instructions. Libraries were sequenced using the Illumina NovaSeq S4 reagent kit (200 cycles) (San Diego, CA, USA). Primers used to generate amplicons from Viral RNA are removed during the tagmentation step of the library preparation protocol. During tagmentation, Amplicons are fragmented and tagged with adapters and bound on to the tagmentation beads. Primers, buffers and other reagents from amplification step are removed during the washes after tagmentation before proceeding with Indexing PCR.

### SARS-CoV-2 genome assembly and multiple sequence alignment

In-house CovSeq pipeline was used in this study, following the best practices and instructions recommended by the Broad Institute’s Genome Analysis ToolKit (GATK)^23^. All CovSeq reads were checked for quality using FastQC software version 0.11.5^24^. Low quality reads, primers and Illumina adapters were removed using Trimmomatric tool version 0.33.0^25^. Trimmed reads were mapped to SARS-CoV-2 reference genome (Wuhan-Hu-1-NC_045512.2/MN908947.3 using Burrows-wheeler Aligner (BWA) v.0.7.12 (BWA-MEM)^26^. The quality check on mapped reads was performed using Qualimap v2.2.1^27^, indicating at least 90% of the reference based mapping exhibited at least tenfold coverage, shown in Supplementary Text 1. Duplicated reads were removed using Picard (v.2.9.4)^28^ and the variant calling was determined using HaplotypeCaller^23^, using a ploidy setting of 1 to account for the haploid genome of the virus. The generated FASTA of each samples were assigned for particular lineages using Pangolin COVID-19 lineage assigner (v 3.1.19) and Nextclade 0.14.1^29,30^, and labeled according to WHO nomenclature.

### Context selection and phylogenetic tree generation

A total of 1,274 SARS-CoV-2 sequences were quality filtered (see below) and used as seed for context selection: a context database suitable for BLAST queries was created using 399,124 SARS-Cov-2 sequences in GISAID as per February 16, 2021.

All local sequences were compared to all GISAID sequences using Nucleotide-Nucleotide BLAST 2.6.0 (blastn)^31^, retaining up to 30 matches per query sequence, with maximum 20 mismatches. Further, a quota of maximal 100 sequences per country is introduced to counter-balance the heterogeneity in national sequencing efforts. The rationale behind this approach is to construct a phylogenetic tree that includes all sequences most relevant to the local samples.

After context selection, 3267 sequences were used to construct the phylogenetic tree using Augur^30^. SARS-CoV-2 Fasta and metadata files were filtered, and aligned to the reference sequence (NC_045512.2/ MN908947.3) using MAFFT v.2^32^, whereas any sequence sites with potential errors were masked^33^. The phylogeny tree was constructed using augur commands tree and refine, which in turn deploy IQ-TREE v1.6.8 and TreeTime^34^. Other augur subcommands were utilized to reconstruct mutations, label clades, and infer geographic movement which can be visualized on Auspice. The entire workflow is managed with snakemake^35^.

### Detection of multiple introductions of VOCs

We repeat the above steps for the subclades of VOCs B.1.1.7 (alpha) and B.1.351 (beta) by selecting all UAE based variants as queries (377 and 39, respectively) contextualized with similar BLAST hits in GISAID sequences according to the pangolin lineage, yielding variant specific contexts with a total of 597 and 237 sequences, respectively. Augur (using TreeTime) also estimates the origin of ancestral nodes, with the caveat that this estimate is skewed towards country-based sequencing efforts. The phylogenies from those extended contexts facilitate the identification of likely local transmission events (and by contrast) international introductions and demonstrate the genetic diversity.

### Phylodynamic analysis

We deploy TreeTime, to plot the history of effective population size, also known as skyline. TreeTime maximizes the coalescence likelihood from a phylogenetic tree similar to other state of the art tools like BEAST^36^, but compares favorably with regard to computational efficiency. We therefore could calculate the effective population size based on all sequences sampled in the UAE, without the need for downsampling. The exact parameter settings are provided in the “Supplementary material”.

### Filtration and samples inclusion

Samples with incomplete demographic data (age, gender, nationality, and patient status) were removed from the analysis. In the combined metadata file comprising local and international samples, the records with missing information such as date of collection were filtered out and not included in the phylogenetic tree analysis. Nextclade 0.14.1 default quality control was adopted in this study for mutation call^30^. Samples that did not pass Nextclade quality check were excluded. The quality control used by Nextclade includes the number missing and ambiguous nucleotides, degree of divergence, and clustered differences. The total number of samples that failed Nextclade quality check were 47. In addition, samples with poor genomic coverage (i.e., coverage < 10×, n = 219) were excluded from this study. In total, two hundred and sixty-four samples were excluded from this study. The final study population that passed filtration and included in the analysis were 1274.

### Severity trait locus mapping

Single-nucleotide mutations of each variant were extracted from the aligned sequences via SNP-sites into a VCF file before converting into PLINK formatted files^37^. The PLINK file was augmented with age and sex information as covariates to model the severity as a binary trait. We deployed regenie (https://rgcgithub.github.io/regenie/) to conduct a whole genome regression on the severity trait^38^. The options set were for modeling binary traits, with a genotype block size of 100, and approximate Firth likelihood ratio test for *p* < 0.01. Further validation was conducted through PLINK’s Assoc command which yields whole genome association analyses with adjusted *p*-values.

### Statistical analysis

The descriptive variables were verified using frequency analysis. Pearson Chi-square test was used to study categorical variables via cross-tabulation. Multivariate logistic regression models tested multivariate relationships between symptom severity and the presence of the variant of concern. Multivariate logistic regression models and whole genome regression with the use of regenie tool was used to test multivariate relationships between symptom severity and the presence of mutations. All regression models accounted for age (continuous) and gender (bivariate: male/female). The significance level adopted for all analyses was p < 0.05. For the mutational analyses, we controlled for multiple testing using the Bonferroni correction for 77 comparisons (number of mutations) to an alpha level of 0.05, resulting in the corrected threshold of 0.05/77 = 6.49 × 10^−4^. All statistical analyses were performed with Statistical Package for Social Science (SPSS) version 20 and R (Version 3.4.1).

## Results

### Patient characteristics

A total of 1274 patients, whose geographical, demographic, and clinical characteristic are shown in Table 1, further stratified by VOC/VOI identification. Participants were recruited from seven emirates across the UAE: 95% from Abu Dhabi, 5% from Sharjah, 0.3% from Ajman, 0.4% Umm Al-Quwain, 0.2% from Ras Al-Khaimah, and 0.2% from Fujairah. Of these cases, 59% were males and 41% were female, with the highest proportion of cases in the > 28 age, and from Middle East (49%) and Asia (41%) group. The majority of patients were home quarantine (81%), whereas 17% were hospitalized and 2% were deceased. Of the 1,274 patients, 460 patients (36.1%) were infected with a SARS-CoV-2 VOC (90.4%) or VOI (9.6%). VOCs include the B.1.1.7 (80.2%, n = 369) B.1.351 (8.0%, n = 37) and P.1 (2.8%, n = 10) variants, whereas the VOIs include the A.23.1 **(**6.9%, n = 32**),** the B.1.525/B.1.526 **(**2.0%, n = 9), and B.1.429 **(**0.2% (3))).

### Overall SARS-CoV-2 lineage distribution in the UAE

The distribution of lineages across the seven emirates in the UAE are shown in Supplementary Fig. 1, with approximately 99 lineages identified. Given that Dubai and Abu Dhabi serve as domestic and global hubs, with one of the largest airports for international passenger traffic, there were a heterogeneous distribution of variants, showing a large proportion of different variants and mutations of interest across the cities. The Northern Emirates (Sharjah, Ajman, Umm Al-Quwain, Ras Al-Khaimah, and Fujairah) had a homogenous distribution of COVID-19 variants, where lineage B.1.1.7 (Yellow; Supplementary Fig. 1) and lineage B.1.351 (Red; Supplementary Fig. 1) dominated the outbreak across the cities.

**Figure 1:**
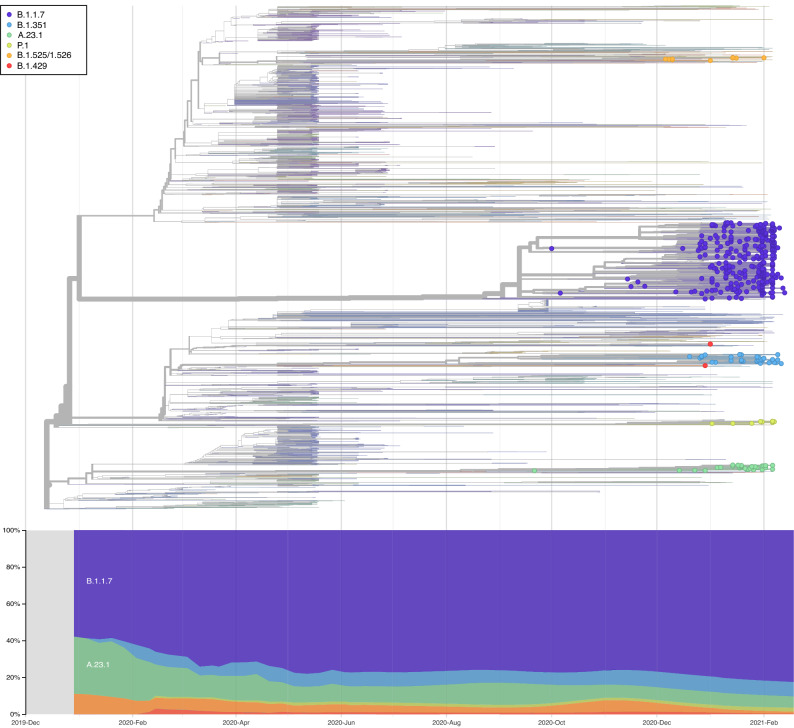
Time-scale Phylogenetic tree of the SARS-CoV-2 lineage in the UAE (shown as circles) from 25 April 2020 to 15 February 2021, contextualized with 1993 most similar sequences selected from GISAID.

A time-scaled phylogeny of 1285 sequences sampled from the UAE, between 25 April 2020 to 15 February 2021 was generated (Supplementary Fig. 2), with 11 major clades and 99 SARS-CoV-2 sub-lineages circulating in the UAE. We observed the presence of B.1.1.263 and B.1.1 in the early months of the pandemic (March 2020 to July 2020). Despite lockdown and strict mitigation measures, phylogenetic branches illustrates sub-clade lineages from multiple genetically-distinct lineages, such as the UK lineages (B.1.1.74, B.1.1.220, B.1.1.296, B.1.1.220 and B.1.1.190), the European lineage (B.1.398 and B.1.1.10) and the UAE lineage (B.1.1.263) between June and July 2020. As the international flight resumed in July 2020, the expansion of B.1.1, B.1.36 and B.1.2 was notable. By October 2020, the major lineages that circulated in the first wave at the beginning of the pandemic were almost completely replaced by worldwide VOCs in a term of few weeks, as demonstrated by Fig. 1. As per our cohort, the first occurrence of the VOCs was B.1.1.7 (9/21/2020), B.1.351 (11/14/2020) and P.1 (12/6/2020), and the first occurrence of the VOIs were A.23.1 (12/14/2020), B.1.525/B.1.526 (11/10/2020), and B.1.429 (12/29/2020). A cluster of sequence (purple; Fig. 1) represents the B.1.1.7, demonstrating the large spread of B.1.1.7 in the UAE. Cluster of B.1.352 (light blue), and P.1 (light orange) appeared between late 2020 to early 2021. The January peak was originated by the VOCs (B.1.1.7, B.1.351 and P.1) and VOI (B.1.525), showing the major dominance of these variants.

Supplementary Fig. 3 demonstrates the daily new confirmed COVID-19 cases (Supplementary Fig. 3A) and daily new deaths (Supplementary Fig. 3B), collected from the official National Crisis and Emergency Management Authority (NCEMA) in the UAE, alongside time in which the mitigation measures were put in place (Supplementary Fig. 3C). As reflected, the number of COVID-19 cases and deaths decreased after strong mitigation measures implemented by the government in March 2020 (Supplementary Fig. 3C). However, shortly after the borders were opened in July 2020, cases started to slowly surge. Supplementary Fig. 3A demonstrates the COVID-19 confirmed cases in the UAE, stratified by the estimated frequency data of VOC vs. Non-VOC. The estimated frequency data of VOC vs. Non-VOC was extrapolated from the sequencing data of this study throughout the time and applied to the NCEMA figures as an estimation analysis. The general wave structure was corroborated through estimation of effective population size including, as demonstrated in Supplementary Fig. 4.

### Patient status and VOC/VOI

Table 2 shows the relationship between patient status and the infection of VOCs/VOIs adjusted by age and gender. Hospitalized status was significantly associated with the patient groups infected with B.1.1.7 (p < 0.001), B.1.351 (p < 0.001) and A.23.1 (p = 0.009). Deceased cases are more likely to occur when infected with B.1.351 (p < 0.001) and A.23.1 (p = 0.022), whereas B.1.1.7 (p = 0.183) was not significantly associated with deceased cases. No significant association was found between patient status and the infection of P.1, B.1.525/526, and B.1.429. When investigating the patient status and the infection of the most common variant in the dataset (B.1.36), patients were less likely to be hospitalized (OR: 0.26 (95% CI 0.13, 0.54), p < 0.001) than the other variants (Supplementary Table 1).

**Table 2:** Association of SARS-CoV-2 VOC/VOI infections to clinical severity status.

	Patient status	Non-B.1.1.7 (n = 787)	B.1.1.7 variant (n = 320)	P-value	Unadjusted OR (95% CI)	Unadjusted P-value	Adjusted OR (95% CI)	Adjusted P-value
B.1.1.7	Home quarantine	84.9% (668)	71.6% (229)	0.001	1.00		1.00	
	Hospitalized	12.0% (100)	27.5% (88)		2.57 (1.85, 3.54)	< 0.001	2.71 (1.86, 3.94)	< 0.001
	Deceased	2.4% (19)	0.9% (3)		0.46 (0.14, 1.57)	0.216	0.43 (0.12, 1.49)	0.183

	Patient status	Non-B.1351 (n = 1074)	B.1.351 variant (n = 33)	P-value	Unadjusted OR (95% CI)	Unadjusted P-value	Adjusted OR (95% CI)	Adjusted P-value
B.1.351	Home quarantine	81.4% (874)	69.7% (23)	0.109	1.00		1.00	
	Hospitalized	16.8% (180)	24.2% (8)		1.68 (0.74, 3.84)	0.211	2.55 (2.13, 3.06)	< 0.001
	Deceased	1.9% (20)	6.1% (2)		3.80 (0.84, 17.25)	0.083	4.26 (2.25, 8.05)	< 0.001

	Patient status	Non-P.1 (n = 1099)	P.1 variant (n = 8)	P-value	Unadjusted OR (95% CI)	Unadjusted P-value	Adjusted OR (95% CI)	Adjusted P-value
P.1	Home quarantine	80.9% (889)	100.0% (8)	0.389	NA			
	Hospitalized	17.1% (188)	0.0% (0)					
	Deceased	2.0% (22)	0.0% (0)					

	Patient status	Non-A.23.1 (n = 1078)	A.23.1 variant (n = 29)	P-value	Unadjusted OR (95% CI)	Unadjusted P-value	Adjusted OR (95% CI)	Adjusted P-value
A.23.1	Home quarantine	81.7% (881)	55.2% (16)	0.001	1.00		1.00	
	Hospitalized	16.4% (177)	37.9% (11)		3.42 (1.56, 7.49)	0.002	3.57 (1.38, 9.22)	0.009
	Deceased	1.9% (20)	6.9% (2)		5.51 (1.19, 25.56)	0.029	7.48 (1.33, 41.97)	0.022

	Patient status	Non-B.1.525/1.526 (n = 1100)	B.1.525/1.526 variant (n = 7)	P-value	Unadjusted OR (95% CI)	Unadjusted P-value	Adjusted OR (95% CI)	Adjusted P-value
B.1.525/1.526	Home quarantine	81.1% (892)	71.4% (5)	0.678	1.00		1.00	
	Hospitalized	16.9% (186)	28.6% (2)		1.92 (0.36, 9.96)	0.438	1.05 (0.60, 0.956)	0.519
	Deceased	2.0% (22)	0.0% (0)		NA	NA	NA	NA

	Patient status	Non-B.1.429 (n = 1105)	B.1.429 variant (n = 2)	P-value	Unadjusted OR (95% CI)	Unadjusted P-value	Adjusted OR (95% CI)	Adjusted P-value
B.1.429	Home quarantine	81.0% (895)	100.0% (2)	0.867	NA			
	Hospitalized	17.0% (188)	0.0% (0)					
	Deceased	2.0% (22)	0.0% (0)					

Chi-squared test of significance was used to measure associations between reference category (Home Quarantine) and each category in the model.

Multivariate analysis (Home Quarantine vs Hospitalized; Home Quarantine vs Deceased) was used for the regression models, presented as unadjusted OR and adjusted OR for age and gender.

NA: a regression analysis was not conducted due to the lack of hospitalized/deceased participants in the case group.

*CI* confidence interval, *NA* not applicable, *OR* odds ratio.

**Figure 2:**
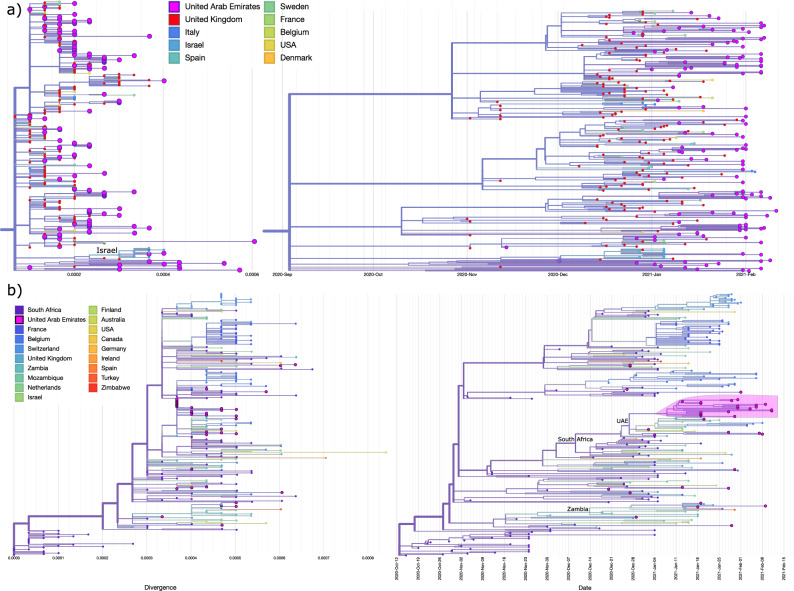
Divergence and time trees for VOCs. (**a**) B.1.1.7/Alpha (**b**) B.1.351/Beta, local cluster highlighted. UAE sequences are contextualized with most similar international sequences as per BLAST search. In both cases, high phylogenetic diversity indicates multiple introductions.

Figure 2 illustrates the phylogeny, divergence and time tree of VOCs (B.1.1.7 and B.1.351) in the UAE. Multiple independent introductions into the country and local transmission clades (i.e. extended transmission chains) are indicated particularly in B.1.1.7 and B.1.351 which explained the high frequencies of VOCs cases in early 2021. The community transmission pattern can be interpreted by limited or no lockdown measures such as in education, social events, and workplace in late 2020 (Supplementary Fig. 3).

The B.1.1.7 variant was identified in 369 cases, with approximately 50 introductions and multiple local transmissions across the UAE, suggesting a widespread local transmission and diversification. The B.1.351 variant was identified in 37 cases, with approximately 15 introductions and one mass spread event infecting 9 cases simultaneously, this spread is highlighted in pink (Fig. 2). However, it should be noted that the quantification the amount of VOC introductions is biased by strongly differing sampling efforts per country and reporting to GISAID. We do however identify a broad phylogenetic diversity, which is highly unlikely to be caused only by local transmissions and based on Augur’s origin estimation for ancestral nodes- the result of multiple international introductions. For some sub-clades (see Fig. 2), we detect intermediate locations (Israel for B.1.1.7 and Zambia for B.1.351), in addition to their country of origin/first detection.

### Emergence of VOCs in the UAE

In our dataset, we identified 2777 different mutations affecting the protein amino acid sequence in the patient sample. The average number of mutations presented in each category were as follows: 14.8 mutation in non-hospitalized and 21.6 in the hospitalized group. Only mutations that are present in 5% of the samples were selected for mutation analysis. The number of mutations based on the above criteria for non-hospitalized, and hospitalized were 35 and 77, respectively. The analysis across all 77 mutations have shown that 37 mutations reached statistical significance after Bonferroni correction at p > 6.49 × 10^−4^, as demonstrated in Supplementary Table 2.

**Figure 3:**
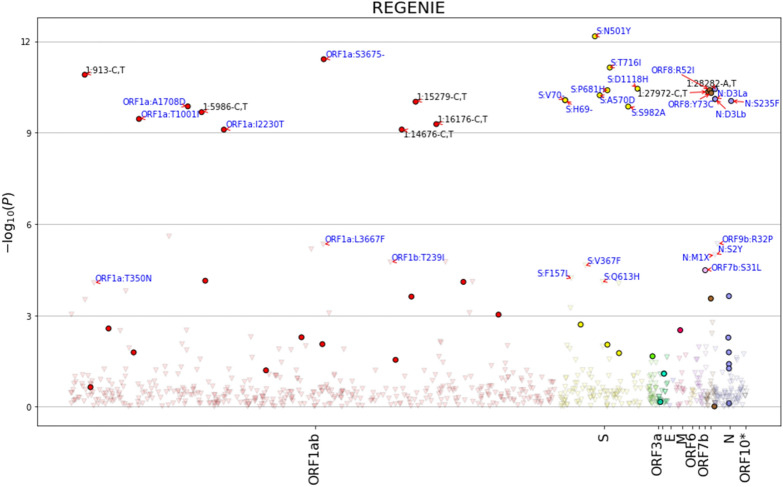
Manhattan plot of regenie’s GWAS on the corresponding SNPs to conduct whole genome regression on the severity trait.

To assess the mutations that are related to hospitalized cases, logistic and ridge regression analysis was conducted on mutations that showed significant association with severity (n = 37). The infection of the mutations adjusted for age and gender were more likely to be associated with hospitalized cases than non-hospitalized (Supplementary Table 2), after Bonferroni correction at p > 6.49 × 10^−4^. The highest proportion of mutations were originated from S and ORF1a genes (35% and 29% respectively). Additional mutations associated with the hospitalized cases of COVID-19 are outlined in Supplementary Table 2. The structural and accessory proteins of SARS-CoV-2 that are significantly associated with hospitalized COVID-19 cases after adjustment for age and gender, and Bonferroni correction at p > 6.49 × 10^−4^, is summarized in Table 3. The complete list of mutations correlated to hospitalized status is presented in Supplementary Table 2. A Manhattan plot (Fig. 3) and the output of regenie's GWAS on the corresponding SNPs (Supplementary Table 3) was generated from the ridge regression analysis were regenie tool was deployed to conduct whole genome regression on the severity trait.

**Table 3:** Brief description of various structural and accessory proteins of SARS-CoV-2 that are significantly associated with hospitalized COVID-19 cases after adjustment for age and gender, and Bonferroni correction at p > 6.49 × 10^−4^.

Protein name	Coding region	Mutations	Role
Spike protein (n = 13)	S	A243, A570D, D1118H, D215G, F157L, H69-V70, N501Y, P681H, Q613H, S982A, T716I, V367F, Y144	Binds to ACE2 host cell receptor and mediates viral entry within the host cell^15,39^
Nucleocapsid protein (n = 6)	N	D3L, M1X, R203K, S194L, S235F, S2Y	Roles in Encapsulates viral nucleic acid^40^
ORF1a (n = 11)	NSP 2	L730F, M372I, T350N	Viral replication, transcription, morphogenesis and evasion of host immune response^41^
	NSP 3	A1708D, I2230T, T1001I	
	NSP 5	K3353	
	NSP 6	F3677, G3676, L3667F, S3675	
ORF1b (n = 2)	NSP 2	T239I	Viral replication, transcription, morphogenesis and evasion of host immune response^41^
	NSP 3	K1383R	
ORF8 (n = 4)	ORF8	K68, Q27, R52I, Y73C	Immune evasion by down-regulating the surface expression of MHC I^42^
ORF9b (n = 1)	ORF9b	R32P	Suppress Interferon response^43^

## Discussion

For the first time, this study demonstrates the entry of the new SARS-CoV-2 variants of concern and interests, and the outbreak dynamics in the UAE. Global massive ongoing transmission and the continuous evolution of new strains demonstrates that strict mitigation measures are important to effectively control the spread of the virus. To do so, a better understanding of the phylogenomic spread and transmission dynamics could contribute to more targeted and effective responses to the pandemic.

The analysis of 1274 viral genomes collected in the UAE, indicates the presence of 11 major clades. The occurrence of the root clades A and East Asian B was clearly seen in the early months of 2020 suggesting early spatiotemporal introduction into the UAE. Distribution of B.1 and B.1.1, which are descendants containing the spike mutation D614G, began in early May 2020 despite the vigilant health measures, which could suggest the multiple independent entry from Europe, Asia, and Middle East prior to the national lockdown. As the nation-wide public health measures were implemented, B.1.1 distributed locally until late July 2020. Despite lockdown and strict measures, we have observed a substantial local transmission within Abu Dhabi and Dubai, in addition to a low frequent spread of sub-clade pangolin lineages such as UK lineages (B.1.1.74, B.1.1.220, B.1.1.296, B.1.1.220 and B.1.1.190), European lineage (B.1.398 and B.1.1.10) and UAE lineage (B.1.1.263) between June and July 2020.

In summer 2020, the expansion of B.1.177 and B.1 in western Europe was notable due to the presence of mutation in RBD (S:S477) which is crucial for ACE2 binding and antibody recognition^15,39^. We reported the presence of these clades in UAE as the international flight resumed in late July 2020, in addition to B.1.1 and B.1.2, which were globally distributed. Strikingly, we observed the resurgence of A clade in early 2021, specifically Pango lineages A, A.20, A.28, A23.1, explaining the possible convergent spike mutations that could confer fitness advantages^44^. We reported the first A.23.1 case in September 2020, and 32 cases in early 2021. Sub-lineages A.22 and A.28 can be explained by independent entry and reported only in home quarantine cases.

The spread of VOCs (B.1.1.7, B.1.351 & P.1) and VOIs (B.1.525) followed the gradual reopening of borders and worldwide travels. Our results indicate that the proportion of VOCs was greater in male than females, and significantly presented in patients aged > 48 years. Concordant to our results^45,46^, B.1.1.7 (p < 0.001), B.1.351 (p < 0.001) and A.23.1 (p = 0.009) were associated with increased severity based on hospitalization rates. Deceased cases are more likely to occur when the patient was infected with B.1.351 (p < 0.001) and A.23.1(p = 0.022), whereas B.1.1.7 (p = 0.183) was not significantly associated with deceased cases. We reported the association between 10 out of 12 defining spike protein substitutions of B.1.1.7 and the hospitalized cases. Similarly, we reported the association between six defining mutations of B.1.351 and hospitalization. For A.23.1, four defining mutations were associated with hospitalized cases. The spread of B.1.351 and B.1.525 lineages have not been associated with hospitalization possibly due to the small size sample.

We have performed mutation analysis to define any significant correlation between patient severity and mutations resulting in amino acids sequence changes. A total of 37 structural and accessory proteins of SARS-CoV-2 are significantly associated with hospitalized COVID-19 cases after adjustment for age and gender, and Bonferroni correction. Overall, we have observed more mutation in the structural spike protein (n = 13). We identified four major mutations of concerns in spike region that are associated with hospitalized cases in our study. N501Y that presents in B.1.1.7 and B.1.351 lineages has been reported to increase ACE2-binding affinity^47^ and as a mean of immune escape^39^. Other mutations such as A570D, D1118H, P681H, S982A, T716I, two deletions H69-V70 and Y144 in spike protein, in addition to D3L, S194L and S235F in nucleocapsid protein were found in B.1.1.7 lineage are in accordance with the studies indicating the high risk of hospital admission and severe disease in B.1.1.7 patients compared to wild-type variant^45,48^. B.1.351 lineages mutations found in this study such as A701V were reported by Campbell et al.^49^ to increase transmissibility by 25% and death in the hospitalized patients by 20%. Other mutations reported in spike and nucleocapsid regions (S: A243; N: M1X and S2Y) in this study have been associated with hospitalized cases, yet no studies have shown any association between these mutations and severity. The importance of ORF1a and ORF1b have been reported in viral replication, transcription, morphogenesis, and evasion of the host of the immune response. Concordant to our results, A1708D, I2230T, and T1001I mutations in ORF1a found in alpha lineages have been associated with hospital admission^45^. The remaining mutations in ORF1ab (L730F, M372I, T350N, A1708D, I2230T, T1001I, K3353, F3677, G3676, L3667F, S3675, T239I, K1383R) have not been reported to correlate to the severity in other studies.

Other significant correlations were reported between hospitalized outcome and accessory proteins such as ORF8 and ORF9b. Although accessory proteins are not involved in virus replication, accumulating evidence demonstrated their critical roles in viral pathogenesis. Most mutations in accessory proteins were at ORF8 which were not identified in other studies. ORF8 was found to induce major histocompatibility class I (MHC1) down-regulation, thus providing protection against cytotoxic T cells (CTLs)^50^. In addition, ORF8 expressing cell and SARS-CoV-2 infected cells are resistant to CLT lysis, which was restored with knockdown of ORF8 expression^50,51^. It is suggested that SARS-CoV-2 could potentially benefit from missense mutations in ORF8 protein to evade immune surveillance^51^. We also identified K68, Q27, R52I, and Y73C mutation in ORF8, and R32P mutation in ORF9b in hospitalized patient. Mutations in ORF9b has been reported to interact with the mitochondria outer membrane protein (TOM70), thus suppresses interferon response^43^.

Limitation of the study should be addressed. At the beginning of pandemic, most patients (asymptomatic and symptomatic) were admitted to the hospital or quarantine areas which could not necessarily reflect the severity of the patient. Therefore, due to the complex nature of the COVID-19 phenotype presentation, statistical and methodological heterogeneity may be present. Also, the admission of patients may be influenced by other factors such as immediate status, comorbidities, and age. Second, the classification of ethnicity might be impression due to using nationality recorded from official passport as a surrogate for ethnicity. Epidemiological features such as travel-related, comorbidities, treatments and severe admission were limited in this study, which impacted post-hoc adjustment analysis. It is clearly noted that 95% of the patients were from the Emirate of Abu Dhabi which indicates the necessity of including further samples from other Emirates. Our mutation analysis may have sampling bias, since only 17% of patients were hospitalized, whereas the remaining were non-hospitalized.

Our study provides an epidemiological insight into the emergence of VOCs and VOIs following borders reopen and worldwide travels. It provides reassurance that hospitalization is markedly more associated with the presence of VOCs. The major strength of this study was the comprehensive longitudinal analysis which covered the early months of COVID-19 in UAE, until the peak of the 3rd wave in February 2021. However, the collection of good quality data such as vaccine status, severity, and travel history in combination with rapid genome sequence are imperative in understanding the behavior and role of variants related to clinical outcomes. This study can contribute to understanding the global transmission of SARS-CoV-2 variants.

## Supplementary Information

Supplementary Information.

## Data Availability

The datasets generated and/or analyzed during the current study are available in the GISAID repository [https://www.epicov.org/epi3/frontend#2306a6] with the corresponding accession IDs (EPI_ISL_431124-431125, 5142492-5142499, 5142501–5142540, 5142543–5142576, 5142581–5142642, 5142644–5142670, 5142673–5142687, 5142690–5142703, 5142707–5142722, 5142724–5142731, 5142739–5142739, 5142754–5142754, 5142763–5142791, 5142795–5142808, 5142813–5142827, 5142830–5142852, 5142856–5142887, 5142889–5142893, 5142895–5142945, 5142950–5142960, 5142965–5142987, 5142991–5143027, 5143032–5143079, 5143082–5143099, 5143101–5143119, 5143122–5143257, 5143259–5143267, 5199363–5199418, 859561–860091).
