# Supplementary material for: Genomic epidemiology and emergence of SARS-CoV-2 variants of concern in the United Arab Emirates
Source: Sci Rep. 2022 Aug 29;12:14669. doi: 10.1038/s41598-022-16967-w (PMC9421632; doi:10.1038/s41598-022-16967-w)
Supplement: Supplementary file 1 — Supplementary Information. [file 41598_2022_16967_MOESM1_ESM.docx]

## **Supplementary Material**

## ***Supplementary Text 1.***

## *Sequence quality control*

The first step of the sequencing pipeline

The trimmomatic command is

java -Xmx32G -jar <TrimmomaticsDir>/trimmomatic-0.36.jar PE -threads 24 -phred33 <FastqFile1> <FastqFile2> <trimOutdir>/<outBasename>.paired.fastq.gz <trimOutdir>/<outBasename>.unpaired.fastq.gz <trimOutdir>/<outRevBasename>.paired.fastq.gz <trimOutdir>/<outRevBasename>.unpaired.fastq.gz ILLUMINACLIP:<illuminaAdapterFile>:2:30:10 LEADING:6 TRAILING:3 SLIDINGWINDOW:4:20 MINLEN:50

*bwa alignment step*

bwa mem –t (number of threads) –R “@RG\tID”${SAMPLE}\ tLB:${SAMPLE}\tSM”${SAMPLE}” /reference genome directory/Ref.fa ${SAMPLE}_Forward.fastq.gz ${SAMPLE}_Reverse.fastq.gz | samtools view -@8 –bs - | samtools sort -@8 - o output.bam

Qualimap step

Qualimap - -java-mem-size=16g bamqc –bam output.bam -outdir qualimap

## *Phylodynamic analysis*

Treetime version 0.8.1 is deployed on an Intel(R) Xeon(R) Gold 6130 CPU @ 2.10GHz machine running Linux Ubuntu 16.04. The complete command line is:

treetime --tree results/tree_raw.nwk --dates metadata.tsv --aln uae.fasta --outdir skyline --coalescent skyline –n-skyline 12

where tree_raw.nwk is the tree generated with augur tree in the augur pipeline, which in turn uses iqtree. The aligned files are 2600 UAE sequences, 1274 from our lab, the remaining 1326 sequences are from GISAID with country being set to United Arab Emirates.

## *Frequency calculation*

We calculate strain frequencies using TreeTime’s kernel density estimation method, with the stiffness parameter set to 3.0.

rule frequencies:

message: "Generating frequencies plot"

input:

tree = rules.refine.output.tree,

metadata = input_metadata

params:

method = "kde", stiffness = 3.0, max_date = "2021-02-14",

pivot_interval = 1, pivot_interval_units = "weeks"

output:

frequencies = "auspice/blastsel_beta3_tip-frequencies.json"

shell:

augur frequencies \

--tree {input.tree} \

--metadata {input.metadata} \

--method {params.method} \

--stiffness {params.stiffness} \

--max-date {params.max_date} \

--pivot-interval {params.pivot_interval} \

--pivot-interval-units {params.pivot_interval_units} \

--output {output.frequencies}


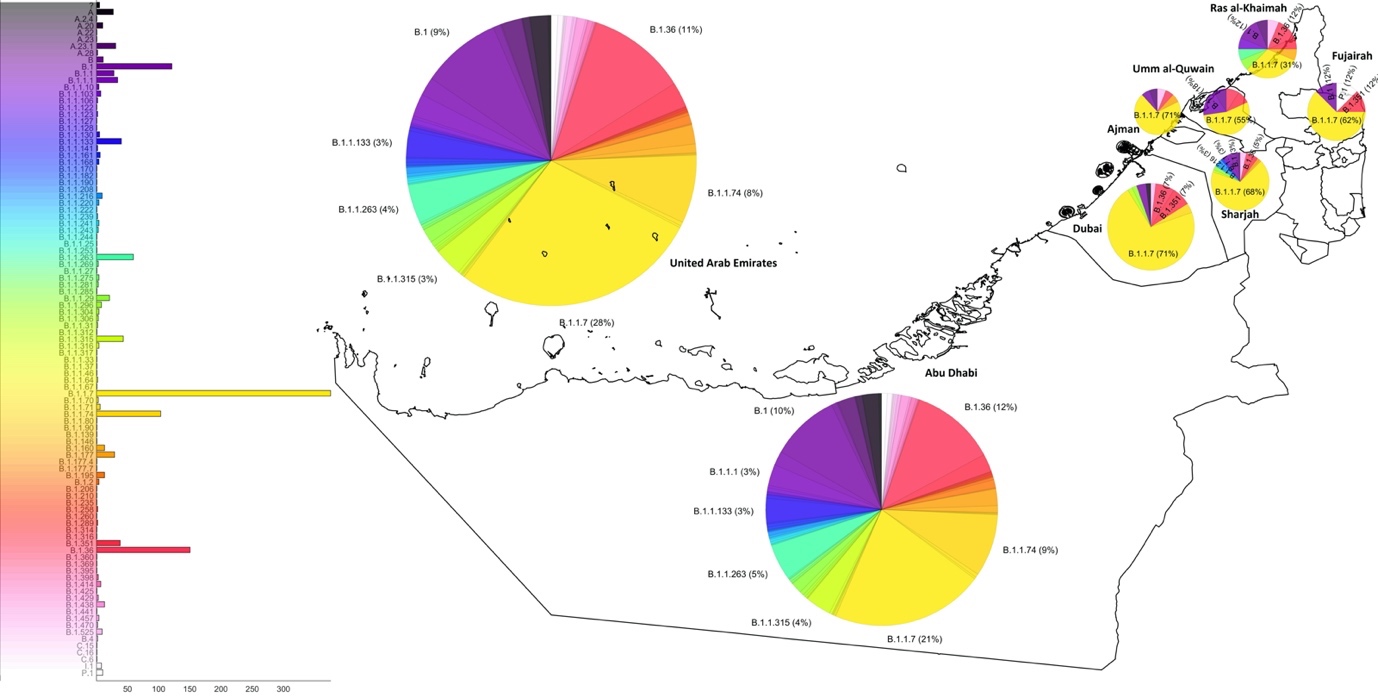


***Supplementary Figure 1. SARS-CoV-2 lineage distribution across the seven Emirates in the UAE from April 25, 2020 to February 15, 2021.***


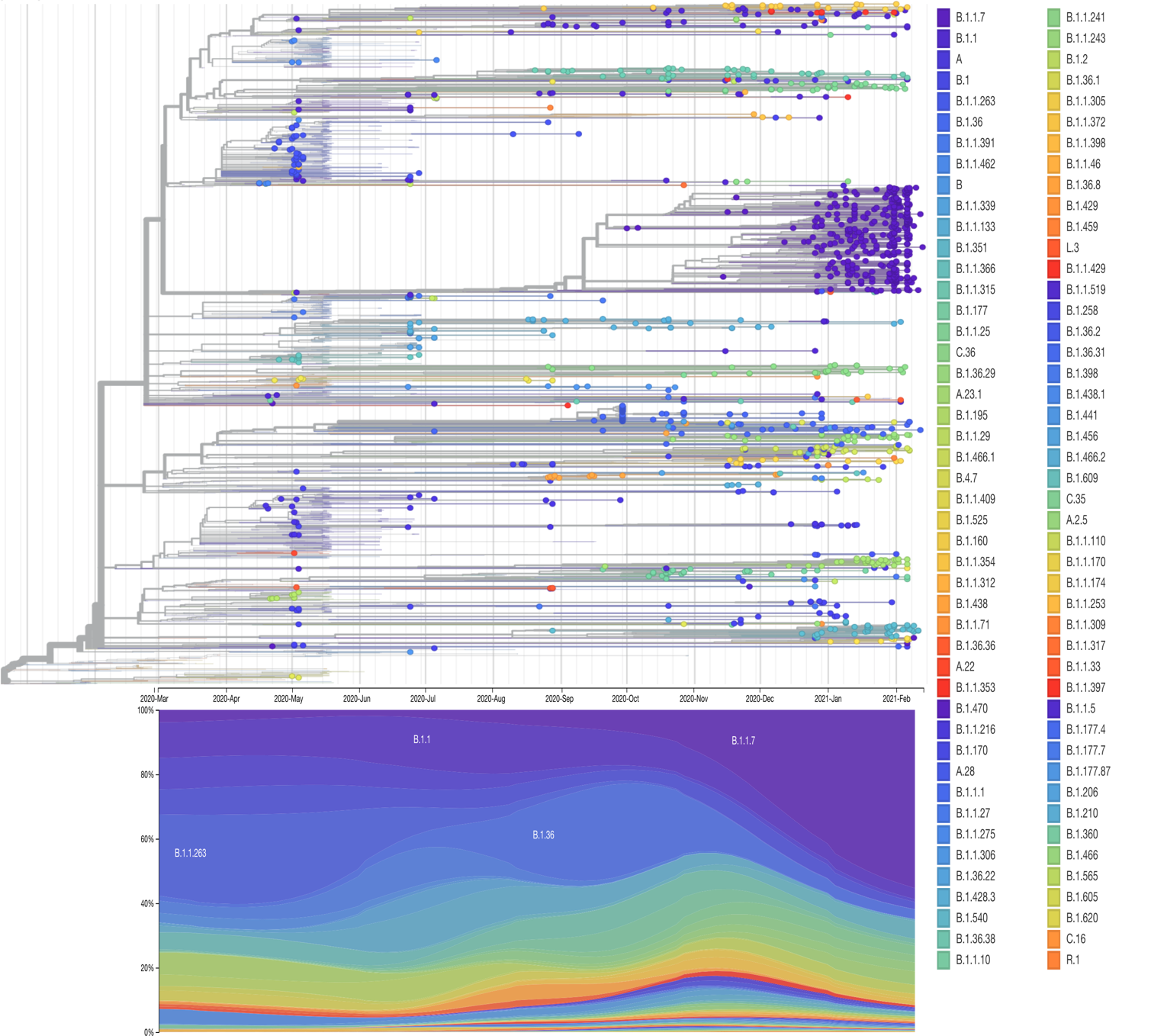


***Supplementary Figure 2. The distribution of 1,285 UAE samples in a contextualized phylogeny comprising in total 3,215 local and global samples. Their respective accumulated pangolin lineage frequencies (as approximated by Kernel Density Estimation using Augur/TreeTime) is shown below and aligned on the same time axis.***


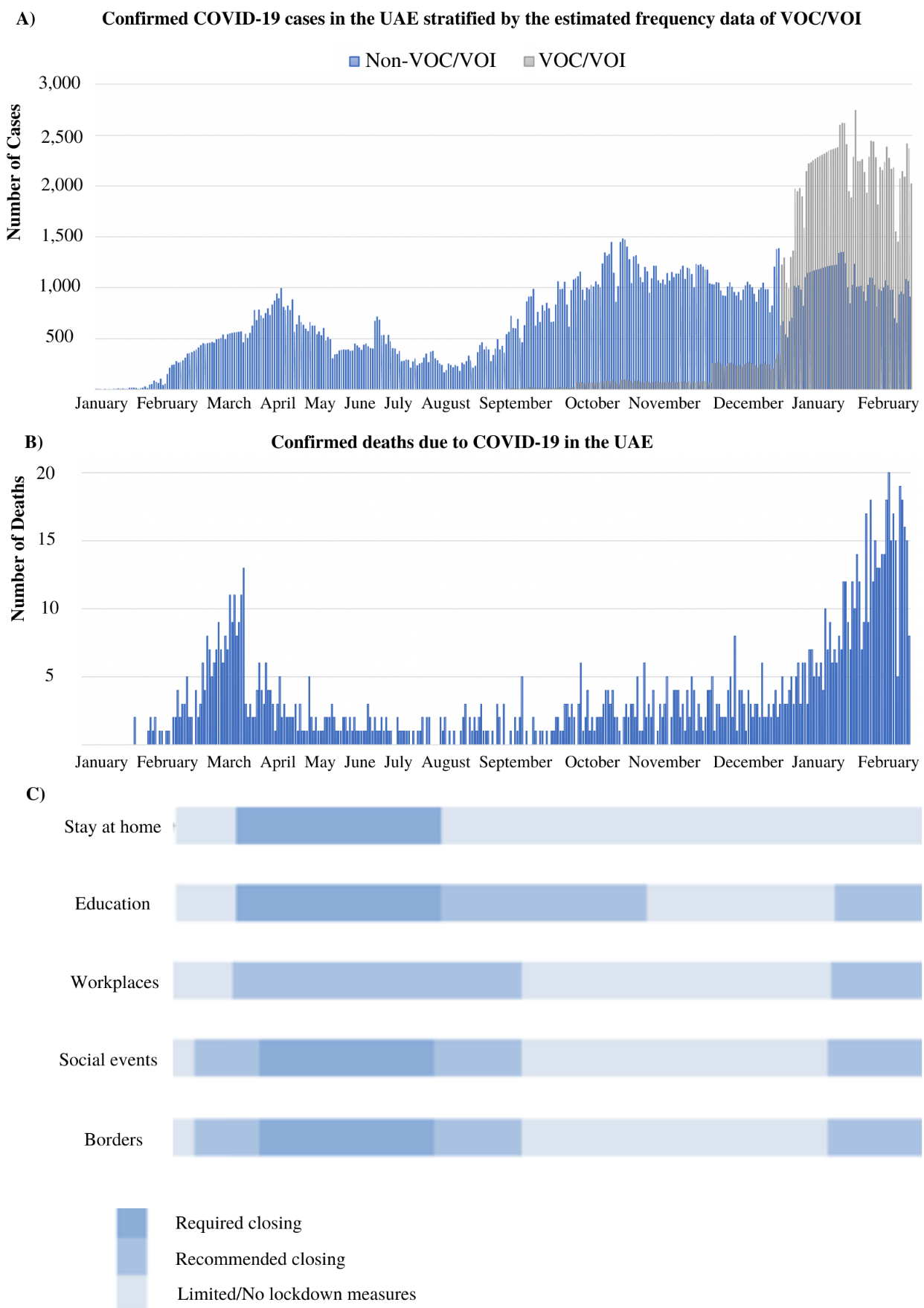


***Supplementary Figure 3. Daily new confirmed COVID-19 cases and daily new deaths, collected from the official National Crisis and Emergency Management Authority (NCEMA) in the UAE, alongside the period of the mitigation measures. (A) COVID-19 confirmed cases in the UAE, stratified by the estimated frequency data of VOC vs. Non-VOC. (B) The total number of confirmed deaths due to COVID-19 in the UAE, collected from the official NCEMA database in the UAE. (C) The time in which mitigation measures were put in place, and the level and intensity of lockdown.***

***Supplementary Figure 4. A) TreeTime based Linear Regression of mutation rate as derived from root-to-tip mutations. B) Skyline plot of Effective Population Size of SARS-CoV-2 in the UAE***
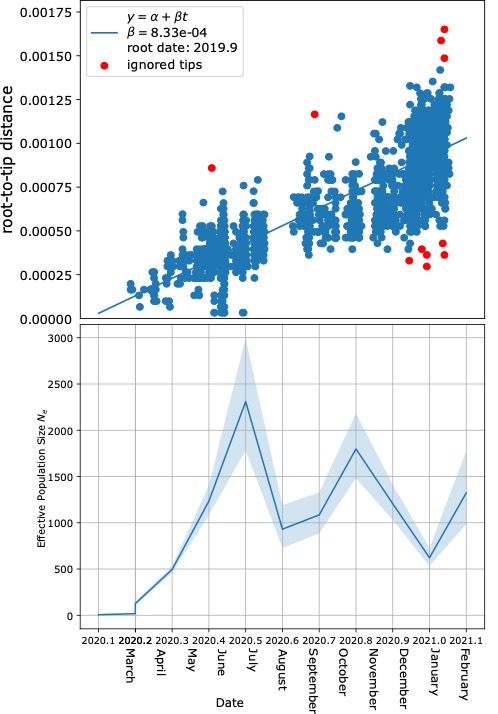
***.***

***Supplementary Table 1. Patient status stratified by the most common variants available in the cohort.***

| B.1.36 | Patient Status | Non-B.1.36 (n=966) | B.1.36 Variant (n=141) | P-Value | Unadjusted OR (95% CI) | Unadjusted P-Value | Adjusted OR (95% CI) | Adjusted P-Value |
| --- | --- | --- | --- | --- | --- | --- | --- | --- |
|  | Home Quarantine  Hospitalized  Deceased | 79.3% (766)  18.5% (179)  2.2% (21) | 92.9% (131)  6.4% (9)  0.7% (1) | **<0.001** | 1.00  0.29 (0.15, 0.59)  0.27 (0.04, 2.08) | **<0.001**  0.214 | 1.00  0.26 (0.13, 0.54)  0.24 (0.03, 1.95) | **<0.001**  0.184 |
| B.1 | **Patient Status** | **Non-B.1**  **(n=1,006)** | **B.1 Variant**  **(n=101)** | **P-Value** | **Unadjusted OR (95% CI)** | **Unadjusted P-Value** | **Adjusted OR (95% CI)** | **Adjusted P-Value** |
|  | Home Quarantine  Hospitalized  Deceased | 81.9% (824)  16.2% (163)  1.9% (19) | 72.3% (73)  24.8% (25)  3.0% (3) | 0.063 | 1.00  1.73 (1.06, 2.81)  1.78 (0.52, 6.16) | 0.026  0.361 | 1.00  1.90 (1.12, 3.23)  2.69 (0.72, 10.13) | **0.018**  **<0.001** |
| B.1.1.74 | **Patient Status** | **Non-B.1.1.74**  **(n=1,057)** | **B.1.1.74 Variant (n=50)** | **P-Value** | **Unadjusted OR (95% CI)** | **Unadjusted P-Value** | **Adjusted OR (95% CI)** | **Adjusted P-Value** |
|  | Home Quarantine  Hospitalized  Deceased | 80.1% (847)  17.8% (188)  2.1% (22) | 100.0% (50)  0.0% (0)  0.0% (0) | 0.002 | NA | | | |
| B.1.1.315 | **Patient Status** | **Non-B.1.1.315 (n=1,070)** | **B.1.1.315 Variant (n=37)** | **P-Value** | **Unadjusted OR (95% CI)** | **Unadjusted P-Value** | **Adjusted OR (95% CI)** | **Adjusted P-Value** |
|  | Home Quarantine  Hospitalized  Deceased | 80.9% (866)  17.2% (184)  1.9% (20) | 83.8% (31)  10.8% (4)  5.4% (2) | **0.208** | 1.00  0.61 (0.21, 1.74)  2.79 (0.63, 12.48) | **0.353**  **0.179** | 1.00  1.07 (1.05, 1.08)  1.11 (1.08, 1.14) | **<0.001**  **<0.001** |
| B.1.1.29 | **Patient Status** | **Non-B.1.1.29 (n=1,065)** | **B.1.1.29 Variant (n=42)** | **P-Value** | **Unadjusted OR (95% CI)** | **Unadjusted P-Value** | **Adjusted OR (95% CI)** | **Adjusted P-Value** |
|  | Home Quarantine  Hospitalized  Deceased | 80.7% (859)  17.3% (184)  2.1% (22) | 90.5% (38)  9.5% (4)  0.0% (0) | 0.249 | 1.00  0.49 (0.17, 1.39)  NA | 0.182  NA | 1.00  0.55 (0.18, 1.62)  NA | 0.278  NA |
| B.1.1.263 | **Patient Status** | **Non- B.1.1.263 (n=1,077)** | **B.1.1.263 Variant (n=30)** | **P-Value** | **Unadjusted OR (95% CI)** | **Unadjusted P-Value** | **Adjusted OR (95% CI)** | **Adjusted P-Value** |
|  | Home Quarantine  Hospitalized  Deceased | 80.5% (867)  17.5% (188)  2.0% (22) | 100.0% (30)  0.0% (0)  0.0% (0) | 0.027 | NA | | | |
| CI, Confidence Interval; NA, Not Applicable; OR, Odds Ratio  Chi-squared test of significance was used to measure associations between reference category (Home Quarantine) and each category in the model.  Multivariate analysis (Home Quarantine vs Hospitalized; Home Quarantine vs Deceased) was used for the regression models, presented as unadjusted OR and adjusted OR for age and gender  NA: a regression analysis was not conducted due to the lack of hospitalized/deceased participants in the case group | | | | | | | | |

**Supplementary Table 2. Association of COVID-19 mutations with patient status (non-hospitalized vs. hospitalized)**

|  | Non-Hospitalized | Hospitalized | P-Value | Unadjusted OR (95% CI) | Unadjusted P-Value | Adjusted OR (95% CI) | Adjusted P-Value |
| --- | --- | --- | --- | --- | --- | --- | --- |
| E:P71L  No  Yes | 894 (83.0%)  25 (69.4%) | 183 (17.0%)  11 (30.6%) | 0.035 | 1.00  2.15 (1.04, 4.44) | 0.039 | 1.00  2.24 (0.96, 5.17) | 0.059 |
| N:D3L  No  Yes | 697 (88.3%)  222 (68.5%) | 92 (11.7%)  102 (31.5%) | <0.001* | 1.00  3.48 (2.52, 4.79) | <0.001* | 1.00  3.84 (2.67, 5.52) | <0.001* |
| N:G204R  No  Yes | 360 (86.1%)  559 (80.4%) | 58 (13.9%)  136 (19.6%) | 0.015 | 1.00  1.51 (1.08, 2.11) | 0.016 | 1.00  1.62 (1.12, 2.33) | 0.010 |
| N:K249R  No  Yes | 892 (82.5%)  27 (84.4%) | 189 (17.5%)  5 (15.6%) | 0.785 | 1.00  0.87 (0.33, 2.29) | 0.785 | 1.00  0.78 (0.27, 2.24) | 0.782 |
| N:M1X  No  Yes | 695 (88.4%)  224 (68.5%) | 91 (11.6%)  103 (31.5%) | <0.001* | 1.00  3.28 (2.21, 5.39) | <0.001* | 1.00  3.88 (2.71, 5.57) | <0.001* |
| N:R203K  No  Yes | 348 (89.0%)  571 (79.1%) | 43 (11.0%)  151 (20.9%) | <0.001* | 1.00  2.14 (1.48, 3.08) | <0.001* | 1.00  2.33 (1.57, 3.47) | <0.001* |
| N:S194L  No  Yes | 873 (83.3%)  46 (70.8%) | 175 (16.7%)  19 (29.2%) | <0.001 | 1.00  0.38 (0.21, 0.68) | <0.001* | 1.00  0.32 (0.17, 0.61) | <0.001* |
| N:S202N  No  Yes | 773 (81.0%)  146 (91.8%) | 181 (19.1%)  13 (8.2%) | 0.010 | 1.00  2.06 (1.18, 3.60) | 0.011 | 1.00  1.99 (1.06, 3.75) | 0.030 |
| N:S235F  No  Yes | 684 (88.6%)  235 (68.9%) | 88 (11.4%)  106 (31.1%) | <0.001* | 1.00  3.51 (2.54, 4.82) | <0.001* | 1.00  3.84 (2.68, 5.51) | <0.001* |
| N:S2Y  No  Yes | 903 (83.7%)  16 (47.1%) | 176 (16.3%)  18 (52.9%) | <0.001* | 1.00  5.77 (2.88, 11.53) | <0.001* | 1.00  4.98 (2.24, 11.05) | <0.001* |
| N:T205I  No  Yes | 851 (82.9%)  68 (79.1%) | 176 (17.1%)  18 (20.9%) | 0.373 | 1.00  1.28 (0.74, 2.20) | 0.370 | 1.00  1.07 (0.58, 1.97) | 0.820 |
| ORF1a:A1708D  No  Yes | 696 (88.3%)  223 (68.6%) | 92 (11.7%)  102 (31.4%) | <0.001* | 1.00  3.46 (2.51, 4.76) | <0.001* | 1.00  3.79 (2.64, 5.45) | <0.001* |
| ORF1a:D1625Y  No  Yes | 917 (82.8%)  2 (33.3%) | 190 (17.2%)  4 (66.7%) | 0.001 | 1.00  9.65 (1.75, 53.01) | 0.009 | 1.00  12.00 (1.78, 80.90) | 0.011 |
| ORF1a:F3677  No  Yes | 663 (89.5%)  256 (68.8%) | 78 (10.5%)  116 (31.2%) | <0.001* | 1.00  3.85 (2.79, 5.31) | <0.001* | 1.00  4.15 (2.89, 5.95) | <0.001* |
| ORF1a:G3676  No  Yes | 663 (89.5%)  256 (68.8%) | 78 (10.5%)  116 (31.2%) | <0.001* | 1.00  3.85 (2.79, 5.31) | <0.001* | 1.00  4.21 (2.94, 6.04) | <0.001* |
| ORF1a:I2230T  No  Yes | 694 (88.0%)  225 (69.4%) | 95 (12.0%)  99 (30.6%) | <0.001* | 1.00  3.21 (2.33, 4.42) | <0.001* | 1.00  3.60 (2.51, 5.16) | <0.001* |
| ORF1a:I300F  No  Yes | 869 (82.8%)  50 (78.1%) | 180 (17.2%)  14 (21.9%) | 0.334 | 1.00  1.35 (0.73, 2.49) | 0.336 | 1.00  1.58 (0.79, 3.17) | 0.192 |
| ORF1a:K1053R  No  Yes | 893 (82.5%)  26 (83.9%) | 189 (17.5%)  5 (16.1%) | 0.846 | 1.00  0.91 (0.34, 2.39) | 0.846 | 1.00  0.81 (0.28, 2.33) | 0.697 |
| ORF1a:K1655N  No  Yes | 891 (83.0%)  28 (71.8%) | 183 (17.0%)  11 (28.2%) | 0.071 | 1.00  1.91 (0.93, 3.91) | 0.076 | 1.00  1.81 (0.80, 4.12) | 0.154 |
| ORF1a:K3353  No  Yes | 880 (83.5%)  39 (66.1%) | 174 (16.5%)  20 (33.9%) | <0.001* | 1.00  2.59 (1.47, 4.55) | <0.001 | 1.00  3.21 (1.67, 6.17) | <0.001* |
| ORF1a:L3201F  No  Yes | 914 (82.9%)  5 (50.0%) | 189 (17.1%)  5 (50.0%) | 0.006 | 1.00  4.83 (1.38, 16.87) | 0.013 | 1.00  5.78 (1.38, 24.05) | 0.016 |
| ORF1a:L3667F  No  Yes | 904 (83.7%)  15 (45.5%) | 176 (16.3%)  18 (54.5%) | <0.001* | 1.00  6.16 (3.04, 12.46) | <0.001* | 1.00  6.51 (2.90, 14.59) | <0.001* |
| ORF1a:L730F  No  Yes | 904 (83.2%)  15 (55.6%) | 182 (16.8%)  12 (44.4%) | <0.001* | 1.00  3.97 (1.83, 8.63) | <0.001 | 1.00  4.60 (1.86, 11.38) | <0.001* |
| ORF1a:M3655I  No  Yes | 879 (83.4%)  40 (67.8%) | 175 (16.6%)  19 (32.2%) | 0.002 | 1.00  2.38 (1.35, 4.21) | 0.003 | 1.00  2.22 (1.16, 4.25) | 0.015 |
| ORF1a:M37521  No  Yes | 897 (83.4%)  22 (57.9%) | 178 (16.6%)  16 (42.1%) | <0.001* | 1.00  3.66 (1.88, 7.11) | <0.001* | 1.00  3.75 (1.75, 8.04) | <0.001* |
| ORF1a:P2018Q  No  Yes | 893 (82.5%)  26 (83.9%) | 189 (17.5%)  5 (16.1%) | 0.846 | 1.00  0.91 (0.34, 2.39) | 0.846 | 1.00  0.697 (0.28, 2.33) | 0.697 |
| ORF1a:S3675  No  Yes | 663 (89.5%)  256 (68.8%) | 78 (10.5%)  116 (31.2%) | <0.001* | 1.00  3.85 (2.79, 5.31) | <0.001* | 1.00  4.15 (2.89, 5.95) | <0.001* |
| ORF1a:T1001I  No  Yes | 673 (88.6%)  246 (69.7%) | 87 (11.4%)  107 (30.3%) | <0.001* | 1.00  3.36 (2.44, 4.62) | <0.001* | 1.00  3.58 (2.51, 5.13) | <0.001* |
| ORF1a:265I  No  Yes | 884 (83.1%)  35 (71.4%) | 180 (16.9%)  14 (28.6%) | 0.036 | 1.00  1.96 (1.04, 3.72) | 0.039 | 1.00  2.07 (1.00, 4.31) | 0.050 |
| ORF1a:T350N  No  Yes | 908 (83.4%)  11 (45.8%) | 181 (16.6%)  13 (54.2%) | <0.001* | 1.00  5.92 (2.61, 13.44) | <0.001* | 1.00  6.53 (2.56, 16.67) | <0.001* |
| ORF1a:V561A  No  Yes | 824 (81.3%)  95 (95.0%) | 189 (18.7%)  5 (5.0%) | <0.001* | 1.00  0.22 (0.09, 0.57) | 0.002 | 1.00  0.21 (0.08, 0.54) | 0.002 |
| ORF1b:A520V  No  Yes | 892 (82.5%)  27 (84.4%) | 189 (17.5%)  5 (15.6%) | 0.785 | 1.00  0.87 (0.33, 2.29) | 0.785 | 1.00  0.77 (0.27, 2.23) | 0.635 |
| ORF1b:K1383R  No  Yes | 890 (83.3%)  29 (65.9%) | 179 (16.7%)  15 (34.1%) | 0.003 | 1.00  2.57 (1.35, 4.89) | 0.004 | 1.00  3.82 (1.83, 7.94) | <0.001* |
| ORF1b:P314L  No  Yes | 57 (74.0%)  862 (83.2%) | 20 (26.0%)  174 (16.8%) | 0.041 | 1.00  0.57 (0.34, 0.98) | 0.043 | 1.00  0.59 (0.32, 1.07) | 0.084 |
| ORF1b:T2165A  No  Yes | 893 (82.5%)  26 (83.9%) | 189 (17.5%)  5 (16.1%) | 0.846 | 1.00  0.91 (0.34, 2.39) | 0.846 | 1.00  0.81 (0.28, 2.33) | 0.697 |
| ORF1b:T239I  No  Yes | 908 (83.5%)  11 (44.0%) | 180 (16.5%)  14 (56.0%) | <0.001* | 1.00  6.42 (2.86, 14.37) | <0.001* | 1.00  7.38 (2.94, 18.55) | <0.001* |
| ORF3a:A54V  No  Yes | 893 (82.5%)  26 (83.9%) | 189 (17.5%)  5 (16.1%) | 0.846 | 1.00  0.91 (0.34, 2.39) | 0.846 | 1.00  0.81 (0.28, 2.33) | 0.697 |
| ORF3a:G172C  No  Yes | 912 (82.8%)  7 (63.6%) | 190 (17.2%)  4 (36.4%) | 0.096 | 1.00  2.74 (0.79, 9.46) | 0.110 | 1.00  3.56 (0.87, 14.43) | 0.076 |
| ORF3a:L108F  No  Yes | 913 (82.8%)  6 (60.0%) | 190 (17.2%)  4 (40.0%) | 0.059 | 1.00  3.20 (0.89, 11.46) | 0.073 | 1.00  4.25 (1.00, 18.04) | 0.049 |
| ORF3a:P240L  No  Yes | 890 (82.5%)  29 (85.3%) | 189 (17.5%)  5 (14.7%) | 0.671 | 1.00  0.81 (0.31, 2.12) | 0.671 | 1.00  0.79 (0.27, 2.31) | 0.674 |
| ORF3a:Q57H  No  Yes | 673 (81.1%)  246 (86.9%) | 157 (18.9%)  37 (13.1%) | 0.025 | 1.00  0.65 (0.44, 0.95) | 0.026 | 1.00  0.0.59 (0.38, .92) | 0.018 |
| ORF3a:S171L  No  Yes | 854 (82.6%)  65 (82.3%) | 180 (17.4%)  14 (17.7%) | 0.944 | 1.00  1.02 (0.56, 1.86) | 0.944 | 1.00  1.00 (0.51, 1.97) | 0.991 |
| ORF3a:T223I  No  Yes | 871 (82.7%)  48 (80.0%) | 182 (17.3%)  12 (20.0%) | 0.590 | 1.00  1.19 (0.62, 2.29) | 0.590 | 1.00  1.70 (0.79, 3.62) | 0.168 |
| ORF3a:W45L  No  Yes | 893 (82.5%)  26 (83.9%) | 189 (17.5%)  5 (16.1%) | 0.846 | 1.00  0.91 (0.34, 2.39) | 0.846 | 1.00  0.85 (0.29, 2.49) | 0.765 |
| ORF7b:E39  No  Yes | 902 (83.1%)  17 (63.0%) | 184 (16.9%)  10 (37.0%) | 0.007 | 1.00  2.88 (1.30, 6.39) | 0.009 | 1.00  3.23 (1.22, 8.52) | 0.018 |
| ORF7b:S31L  No  Yes | 839 (81.2%)  80 (100.0%) | 194 (18.8%)  0 (0.0%) | <0.001* | N/A | | | |
| ORF8:E92K  No  Yes | 888 (83.5%)  31 (63.3%) | 176 (16.5%)  18 (36.7%) | <0.001* | 1.00  2.93 (1.60, 5.35) | <0.001* | 1.00  2.85 (1.46, 5.57) | <0.001 |
| ORF8:K68  No  Yes | 799 (85.4%)  120 (67.8%) | 137 (14.6%)  57 (32.2%) | <0.001* | 1.00  2.77 (1.93, 3.98) | <0.001* | 1.00  2.38 (1.56, 3.62) | <0.001* |
| ORF8:L84S  No  Yes | 882 (83.4%)  37 (67.3%) | 176 (16.6%)  18 (32.7%) | 0.002 | 1.00  2.44 (1.35, 4.38) | 0.003 | 1.00  2.61 (1.35, 5.02) | 0.004 |
| ORF8:Q27  No  Yes | 693 (88.5%)  226 (68.5%) | 90 (11.5%)  104 (31.5%) | <0.001* | 1.00  3.54 (2.57, 4.87) | <0.001* | 1.00  3.69 (2.56, 5.32) | <0.001* |
| ORF8:R52I  No  Yes | 694 (88.5%)  225 (68.4%) | 90 (11.5%)  104 (31.6%) | <0.001* | 1.00  3.72 (2.57, 5.35) | <0.001* | 1.00  3.71 (2.58, 5.35) | <0.001* |
| ORF8:Y73C  No  Yes | 694 (88.5%)  225 (68.4%) | 90 (11.5%)  104 (31.6%) | <0.001* | 1.00  3.56 (2.58, 4.91) | <0.001* | 1.00  3.67 (2.55, 5.28) | <0.001* |
| ORF9b:P3L  No  Yes | 906 (82.7%)  13 (76.5%) | 190 (17.3%)  4 (23.5%) | 0.504 | 1.00  1.46 (0.47, 4.55) | 0.509 | 1.00  1.21 (0.31, 4.76) | 0.782 |
| ORF9b:R32P  No  Yes | 904 (83.7%)  15 (45.5%) | 176 (16.3%)  18 (54.5%) | <0.001* | 1.00  6.16 (3.05, 12.46) | <0.001* | 1.00  6.27 (2.83, 13.92) | <0.001* |
| S:A243  No  Yes | 894 (83.0%)  25 (69.4%) | 183 (17.0%)  11 (30.6%) | 0.035 | 1.00  2.15 (1.04, 4.45) | 0.039 | 1.00  2.17 (0.93, 5.07) | 0.072 |
| S:A570D  No  Yes | 697 (88.5%)  222 (68.3%) | 91 (11.5%)  103 (31.7%) | <0.001* | 1.00  3.55 (2.58, 4.89) | <0.001* | 1.00  3.67 (2.55, 5.30) | <0.001* |
| S:A701V  No  Yes | 892 (83.1%)  27 (69.2%) | 182 (16.9%)  12 (30.8%) | 0.025 | 1.00  2.17 (1.08, 4.38) | 0.029 | 1.00  2.33 (1.04, 5.25) | 0.040 |
| S:D1118H  No  Yes | 697 (88.5%)  222 (68.3%) | 91 (11.5%)  103 (31.7%) | <0.001* | 1.00  3.55 (2.58, 4.89) | <0.001* | 1.00  3.73 (2.58, 5.38) | <0.001* |
| S:D215G  No  Yes | 894 (83.0%)  25 (69.4%) | 183 (17.0%)  11 (30.6%) | 0.035 | 1.00  2.15 (1.04, 4.46) | 0.072 | 1.00  2.17 (0.93, 5.06) | 0.072 |
| S:D614G  No  Yes | 38 (69.1%)  881 (83.3%) | 17 (30.9%)  177 (16.7%) | 0.007 | 1.00  0.45 (0.25, 0.81) | 0.008 | 1.00  0.44 (0.23, 0.85) | <0.001 |
| S:D80A  No  Yes | 891 (83.0%)  28 (70.0%) | 182 (17.0%)  12 (30.0%) | 0.033 | 1.00  2.09 (1.05, 4.20) | 0.037 | 1.00  2.28 (1.04, 5.05) | 0.041 |
| S:E484K  No  Yes | 887 (83.0%)  32 (72.7%) | 182 (17.0%)  12 (27.3%) | 0.079 | 1.00  1.83 (0.92, 3.62) | 0.083 | 1.00  1.61 (0.74, 3.51) | 0.227 |
| S:F157L  No  Yes | 904 (83.5%)  15 (48.4%) | 178 (16.5%)  16 (51.6%) | <0.001* | 1.00  5.42 (2.63, 11.15) | <0.001* | 1,00  5.49 (2.38, 12.62) | <0.001* |
| S:H245  No  Yes | 894 (83.0%)  25 (69.4%) | 183 (17.0%)  11 (30.6%) | 0.035 | 1.00  2.15 (1.04, 4.45) | 0.039 | 1.00  2.24 (0.98, 5.15) | 0.057 |
| S:H69-V70  No  Yes | 691 (88.5%)  228 (68.7%) | 90 (11.5%)  104 (31.3%) | <0.001* | 1.00  3.50 (2.55, 4.82) | <0.001* | 1.00  3.78 (2.64, 5.43) | <0.001* |
| S:K417N  No  Yes | 896 (83.1%)  23 (65.7%) | 182 (16.9%)  12 (34.3%) | 0.008 | 1.00  2.57 (1.25, 5.26) | 0.010 | 1.00  2.35 (1.03, 5.38) | 0.042 |
| S:L242H  No  Yes | 894 (83.0%)  25 (69.4%) | 183 (17.0%)  11 (30.6%) | 0.035 | 1.00  2.15 (1.04, 4.45) | 0.039 | 1.00  2.24 (0.97, 5.15) | 0.057 |
| S:L244  No  Yes | 894 (83.0%)  25 (69.4%) | 183 (17.0%)  11 (30.6%) | 0.035 | 1.00  2.15 (1.04, 4.45) | 0.039 | 1.00  2.24 (0.97, 5.15) | 0.057 |
| S:N439K  No  Yes | 891 (82.6%)  28 (82.4%) | 188 (17.4%)  6 (17.6%) | 0.973 | 1.00  1.01 (0.41, 2.48) | 0.973 | 1.00  1.05 (0.38, 2.83) | 0.928 |
| S:N501Y  No  Yes | 667 (89.7%)  252 (68.3%) | 77 (10.3%)  117 (31.7%) | <0.001* | 1.00  4.02 (2.91, 5.50) | <0.001* | 1.00  4.25 (2.94, 6.13) | <0.001* |
| S:P681H  No  Yes | 694 (88.4%)  225 (68.6%) | 91 (11.6%)  103 (31.4%) | <0.001* | 1.00  3.49 (2.53, 4.81) | <0.001* | 1.00  3.73 (2.58, 5.38) | <0.001* |
| S:P681R  No  Yes | 882 (83.5%)  37 (64.9%) | 174 (16.5%)  20 (35.1%) | <0.001* | 1.00  2.74 (1.55, 4.83) | 0.001 | 1.00  2.58 (1.34, 4.98) | 0.004 |
| S:Q613H  No  Yes | 903 (83.5%)  16 (50.0%) | 178 (16.5%)  16 (50.0%) | <0.001* | 1.00  5.07 (2.49, 10.33) | <0.001* | 1.00  4.93 (2.19, 11.06) | <0.001* |
| S:S982A  No  Yes | 692 (88.4%)  227 (68.8%) | 91 (11.6%)  103 (31.2%) | <0.001* | 1.00  3.45 (2.52, 4.75) | <0.001* | 1.00  3.58 (2.48, 5.15) | <0.001* |
| S:T716I  No  Yes | 695 (88.6%)  224 (68.1%) | 89 (11.4%)  105 (31.9%) | <0.001* | 1.00  3.66 (2.65, 5.04) | <0.001* | 1.00  4.88 (2.69, 5.61) | <0.001* |
| S:V367F  No  Yes | 904 (83.5%)  15 (48.4%) | 178 (16.5%)  16 (51.6%) | <0.001* | 1.00  5.41 (2.63, 11.15) | <0.001* | 1.00  5.71 (2.59, 12.95) | <0.001* |
| S:Y144  No  Yes | 689 (88.6%)  230 (68.7%) | 89 (11.4%)  105 (31.3%) | <0.001* | 1.00  3.53 (2.56, 4.86) | <0.001* | 1.00  3.56 (2.47, 5.12) | <0.001* |
| CI, Confidence Interval; NA, Not Applicable; OR, Odds Ratio  Chi-squared test of significance was used to measure associations between reference category (Home Quarantine) and each category in the model.  Multivariate analysis (non-Hospitalized vs Hospitalized) was used for the regression models, presented as unadjusted OR and adjusted OR for age and gender  NA: a regression analysis was not conducted due to the lack of hospitalized/deceased participants in the case group  P-values significant at a Bonferroni-corrected level of 0.000649 are marked with an asterisk | | | | | | | |

***Supplementary Table 3. Ridge regression output of regenie's GWAS on the corresponding SNPs, and the P values for the mutations that were analyzed in Table 3.***

| **AA Mutation** | **NT Mutation** | **Type** | **Statistical P-value** | **Regenie -log10P** |
| --- | --- | --- | --- | --- |
| **S:V70** | 1:21769-*,T | INDEL | <0.001* | 10.068 |
| **S:V367F** | 1:22661-G,T | SNP | <0.001* | 4.63754 |
| **S:T716I** | 1:23709-C,T | SNP | <0.001* | 11.1377 |
| **S:S982A** | 1:24506-G,T | SNP | <0.001* | 9.85706 |
| **S:Q613H** | 1:23401-G,T | SNP | <0.001* | 4.10231 |
| **S:P681R** | 1:23604-C,G | SNP | <0.001* | 2.0382 |
| **S:P681H** | 1:23604-A,C | SNP | <0.001* | 10.3931 |
| **S:N501Y** | 1:23063-A,T | SNP | <0.001* | 12.1621 |
| **S:N439K** | 1:22879-A,C | SNP | 0.973 | 0.0318822 |
| **S:K417N** | 1:22813-G,T | SNP | 0.008 | 1.05407 |
| **S:H69** | 1:21767-*,C | INDEL | <0.001* | 10.068 |
| **S:F157L** | 1:22033-A,C | SNP | <0.001* | 4.21943 |
| **S:E484K** | 1:23012-A,G | SNP | 0.079 | 0.367331 |
| **S:D80A** | 1:21801-A,C | SNP | 0.033 | 0.929815 |
| **S:D614G** | 1:23403-A,G | SNP | 0.007 | 1.97982 |
| **S:D215G** | 1:22206-A,G | SNP | 0.035 | 0.7805 |
| **S:D1118H** | 1:24914-C,G | SNP | <0.001* | 10.4413 |
| **S:A701V** | 1:23664-C,T | SNP | 0.025 | 1.01566 |
| **S:A570D** | 1:23271-A,C | SNP | <0.001* | 10.2274 |
| **ORF9b:R32P** | 1:28378-C,G | SNP | <0.001* | 5.34289 |
| **ORF9b:P3L** | 1:28291-C,T | SNP | 0.504 | 0.0457688 |
| **ORF8:Y73C** | 1:28111-A,G | SNP | <0.001* | 10.2999 |
| **ORF8:R52I** | 1:28048-G,T | SNP | <0.001* | 10.4045 |
| **ORF8:L84S** | 1:28144-C,T | SNP | 0.002 | 2.40082 |
| **ORF8:E92K** | 1:28167-A,G | SNP | <0.001* | 2.75803 |
| **ORF7b:S31L** | 1:27847-C,T | SNP | <0.001* | 4.49415 |
| **ORF3a:W45L** | 1:25526-G,T | SNP | 0.846 | 0.111391 |
| **ORF3a:T223I** | 1:26060-C,T | SNP | 0.59 | 1.08712 |
| **ORF3a:S171L** | 1:25904-C,T | SNP | 0.944 | 0.160542 |
| **ORF3a:Q57H** | 1:25563-G,T | SNP | 0.025 | 1.65804 |
| **ORF3a:P240L** | 1:26111-C,T | SNP | 0.671 | 0.155975 |
| **ORF3a:L108F** | 1:25714-C,T | SNP | 0.059 | 1.44556 |
| **ORF3a:G172C** | 1:25906-G,T | SNP | 0.096 | 1.06519 |
| **ORF3a:A54V** | 1:25553-C,T | SNP | 0.846 | 0.111391 |
| **ORF1b:T239I** | 1:14183-C,T | SNP | <0.001* | 4.74458 |
| **ORF1b:T2165A** | 1:19960-A,G | SNP | 0.846 | 0.111391 |
| **ORF1b:P314L** | 1:14408-C,T | SNP | 0.041 | 1.54053 |
| **ORF1b:K1383R** | 1:17615-A,G | SNP | 0.003 | 3.08145 |
| **ORF1b:A520V** | 1:15026-C,T | SNP | 0.785 | 0.13487 |
| **ORF1a:V561A** | 1:1947-C,T | SNP | <0.001* | 2.57055 |
| **ORF1a:T350N** | 1:1314-A,C | SNP | <0.001* | 4.06587 |
| **ORF1a:T1001I** | 1:3267-C,T | SNP | <0.001* | 9.45005 |
| **ORF1a:S3675** | 1:11289-*,C | INDEL | <0.001* | 11.4108 |
| **ORF1a:P2018Q** | 1:6318-A,C | SNP | 0.846 | 0.111391 |
| **ORF1a:M37521** | 1:11521-G,T | SNP | <0.001* | 3.07147 |
| **ORF1a:M3655I** | 1:11230-G,T | SNP | 0.002 | 2.05614 |
| **ORF1a:L730F** | 1:2453-C,T | SNP | <0.001* | 2.49121 |
| **ORF1a:L3667F** | 1:11266-G,T | SNP | <0.001* | 5.3295 |
| **ORF1a:L3201F** | 1:9866-C,T | SNP | 0.006 | 1.86633 |
| **ORF1a:K1655N** | 1:5230-G,T | SNP | 0.071 | 1.19369 |
| **ORF1a:K1053R** | 1:3423-A,G | SNP | 0.846 | 0.111391 |
| **ORF1a:I300F** | 1:1163-A,T | SNP | 0.334 | 0.644825 |
| **ORF1a:I2230T** | 1:6954-C,T | SNP | <0.001* | 9.09876 |
| **ORF1a:D1625Y** | 1:5138-G,T | SNP | 0.001 | 2.05611 |
| **ORF1a:A1708D** | 1:5388-A,C | SNP | <0.001* | 9.86231 |
| **N:T205I** | 1:28887-C,T | SNP | 0.373 | 0.111916 |
| **N:S2Y** | 1:28278-A,C | SNP | <0.001* | 4.98616 |
| **N:S235F** | 1:28977-C,T | SNP | <0.001* | 10.039 |
| **N:S202N** | 1:28878-A,G | SNP | 0.01 | 1.78844 |
| **N:S194L** | 1:28854-C,T | SNP | <0.001* | 2.27178 |
| **N:R203K** | 1:28881-A,G | SNP | <0.001* | 3.63284 |
| **N:M1X** | 1:28278-A,C | SNP | <0.001* | 4.98616 |
| **N:K249R** | 1:29019-A,G | SNP | 0.785 | 0.129669 |
| **N:G204R** | 1:28883-C,G | SNP | 0.015 | 1.25881 |
| **N:D3L** | 1:28280-C,G | SNP | <0.001* | 10.1046 |
| **E:P71L** | 1:26456-C,T | SNP | 0.035 | 0.856647 |
| **P-values significant at a Bonferroni-corrected level of 0.000649 are marked with an asterisk** | | | | |
